# Neutrophil-to-lymphocyte ratio in amyotrophic lateral sclerosis: a systematic review and meta-analysis

**DOI:** 10.1093/braincomms/fcag132

**Published:** 2026-04-11

**Authors:** Negin Eissazade, Lena Nazemi, Bahram Haghi Ashtiani, Maziar Moradi-Lakeh

**Affiliations:** Cellular and Molecular Research Center, Iran University of Medical Sciences, Tehran 1388319967, Iran; Brain and Cognition Clinic, Institute for Cognitive Sciences Studies, Tehran 1594834111, Iran; Department of Neurology, Rasool Akram Hospital, School of Medicine, Iran University of Medical Sciences, Tehran 1445613131, Iran; Department of Neurology, Firoozgar Hospital, Iran University of Medical Sciences, Tehran 1593748711, Iran; Gastrointestinal and Liver Diseases Research Center, Iran University of Medical Sciences, Tehran 233423445, Iran

**Keywords:** amyotrophic lateral sclerosis, neutrophil-to-lymphocyte ratio, neutrophils, lymphocytes

## Abstract

Amyotrophic lateral sclerosis (ALS) is a fatal neurodegenerative disease with limited diagnostic and prognostic biomarkers. The neutrophil-to-lymphocyte ratio (NLR), a marker of systemic inflammation, has been proposed as a potential indicator. This systematic review and meta-analysis assesses the diagnostic and prognostic value of NLR in ALS. We searched PubMed, Scopus, Embase and Web of Science through June 2025 for peer-reviewed studies evaluating NLR in adults with ALS diagnosed by established criteria. Eligible studies reported validated measurements of NLR and diagnostic or prognostic outcomes. Two reviewers independently extracted data and assessed quality. Random-effects meta-analyses were performed, with heterogeneity, publication bias, evidence certainty and sources of heterogeneity evaluated using meta-regression. Sixteen studies from 12 countries including 357 044 participants met inclusion criteria, comprising 8710 ALS patients (mean age 60.3 years; 59.1% male) and 348 334 controls (mean age 57.8 years; 47.6% male). Meta-analysis of 11 studies showed a pooled mean NLR of 2.74 in ALS patients [95% CI (2.42, 3.10); *I*^2^ = 95.4%], while three control studies yielded a pooled mean NLR of 1.94 [95% CI (1.55, 2.43); *I*^2^ = 94.7%]. Comparison of three studies demonstrated a 35% higher NLR in ALS patients than controls [95% CI (1.03, 1.76); *I*^2^ = 88.3%], with low certainty according to GRADE due to observational design and substantial heterogeneity. Elevated NLR was consistently associated with worse clinical outcomes, including faster disease progression, lower ALSFRS-r scores, reduced forced vital capacity, shorter survival and increased mortality. Pooled univariate analyses from four studies showed that higher NLR predicted mortality [HR = 1.16; 95% CI (1.04, 1.29); *I*^2^ = 93.8%]. Multivariable-adjusted analyses from six studies confirmed NLR as an independent predictor of poorer survival (HR = 1.13; 95% CI (1.06, 1.21); *I*^2^ = 86.5%), with heterogeneity modestly reduced after adjustment for age and sample size. Certainty of evidence for prognostic outcomes was rated low to moderate. Associations between higher NLR and age at onset, sex and classical ALS phenotype were inconsistent. NLR correlated with inflammatory markers and gut microbiota features, supporting a potential mechanistic link between systemic inflammation and ALS disease progression. Elevated NLR is associated with ALS diagnosis and poorer prognosis, including faster disease progression and reduced survival. Despite heterogeneity and potential bias, NLR appears to be a readily accessible biomarker for disease monitoring and risk stratification in ALS, warranting validation in large, longitudinal studies.

## Teaser

Eissazade *et al*. report that elevated neutrophil-to-lymphocyte ratio (NLR) is associated with faster disease progression, reduced survival and higher mortality in amyotrophic lateral sclerosis. Their findings suggest NLR may serve as a readily accessible biomarker reflecting systemic inflammation and disease activity, although further longitudinal studies are needed to confirm its independent prognostic value.

## Graphical Abstract

**Graphical Abstract:**
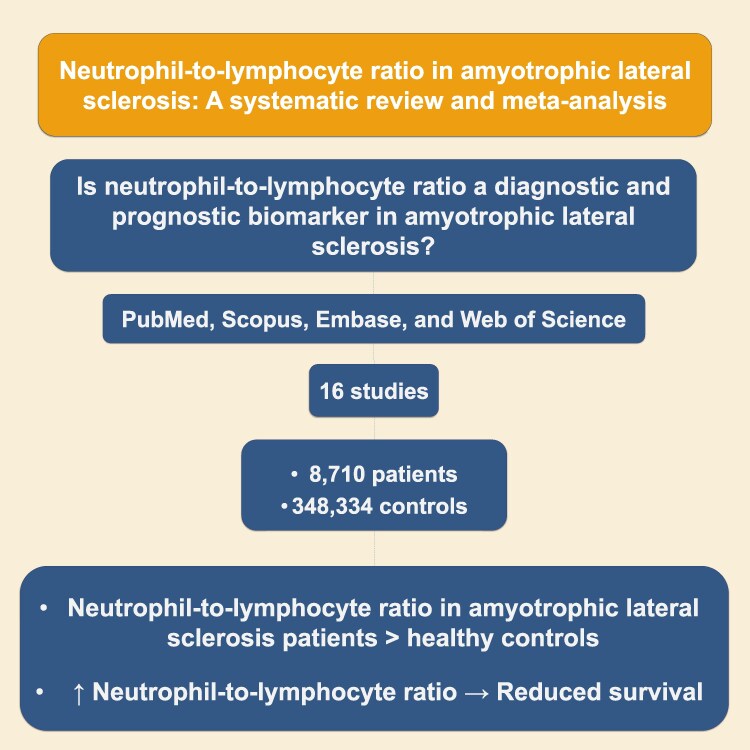

## Introduction

Amyotrophic lateral sclerosis (ALS) is a rare neurodegenerative disorder characterized by the progressive degeneration of upper and lower motor neurons, resulting in muscle weakness, paralysis, respiratory failure and ultimately death. The median survival time following diagnosis is typically 3–5 years,^1^ underscoring the critical need for reliable prognostic markers to better predict disease progression. Historically, prognosis has been based primarily on clinical parameters such as age of onset, diagnostic classification according to the revised El Escorial criteria, Gold Coast criteria, disease phenotype, site of onset, body mass index (BMI), ALS Functional Rating Scale-Revised (ALSFRS-r) score and forced vital capacity (FVC).^2-5^ In recent years, research efforts have increasingly focused on identifying novel biomarkers that may offer additional prognostic value. Frequently investigated biomarkers include albumin, C-reactive protein (CRP), ferritin, creatinine, uric acid, haemoglobin, various electrolytes (potassium, sodium, calcium), glucose and neurofilament light chain (NfL).^6-12^

Emerging evidence from both human and animal studies indicates that neuroinflammation plays a pivotal role in the pathophysiology of ALS, with immune activation and inflammatory processes that contribute to neuronal injury and accelerate disease progression.^13,14^ A deeper understanding of the inflammatory pathways involved in ALS is critical for developing targeted therapeutic strategies aimed at modulating immune responses and slowing disease advancement. Among the various biomarkers of systemic inflammation, the neutrophil-to-lymphocyte ratio (NLR) has gained attention as a simple, inexpensive and easily obtainable parameter derived from routine complete blood count analysis. Elevated NLR has been associated with adverse outcomes in a range of conditions, including diabetes, chronic kidney disease, heart failure, hypertension, coronary artery disease and numerous malignancies.^15,16^ More recently, a growing body of research has suggested a potential association between increased NLR and reduced survival in patients with ALS.^17-30^

However, findings across studies have been inconsistent. Therefore, we conducted this systematic review and meta-analysis to elucidate the diagnostic and prognostic significance of NLR in ALS, with the goal of enhancing our understanding of the role of inflammation in ALS pathogenesis and identifying potential biomarkers for disease monitoring and prognostication.

## Materials and methods

### Protocol and registration

This study adheres to the Preferred Reporting Items for Systematic Reviews and Meta-Analyses (PRISMA) guidelines.^31^ The protocol was registered in the International Prospective Register of Systematic Reviews (PROSPERO) (ID: CRD42024536668).

### Eligibility criteria

Inclusion criteria were defined using the PICOT framework (Population, Intervention, Comparator, Outcomes, Timeframe). Studies were eligible if they included adult patients diagnosed with ALS based on the El Escorial criteria^32^ or equivalent diagnostic standards. The primary variable of interest was the NLR, assessed for its diagnostic and/or prognostic relevance. Comparators included healthy controls or individuals without neurodegenerative or inflammatory disorders. The outcome of interest was the ability of NLR to distinguish ALS from non-ALS populations, as well as its prognostic association with disease progression or survival, based on data reported at baseline or during follow-up, without restriction on follow-up duration. Secondary outcomes included the correlation of higher NLR with various clinical and demographic factors assessed across studies.

Only original, peer-reviewed research articles published in English were considered eligible for inclusion. Eligible study designs included observational studies (cross-sectional, case-control and cohort studies) that provided sufficient quantitative data for analysis. Exclusion criteria encompassed review articles, meta-analyses, conference abstracts, editorials, letter to the editor, study protocols and any studies that lacked original data. Studies with missing or incomplete data on essential variables were excluded from the analysis.

### Information sources, search strategy and study selection

A comprehensive literature search was conducted in June 2025 across PubMed, Scopus, Embase and Web of Science, without date restrictions. The search strategy employed keywords such as ‘*neutrophil-to-lymphocyte ratio*’ and ‘*amyotrophic lateral sclerosis*’. Full search details are provided in Supplementary Material 1.

An initial screening of titles and abstracts was conducted to exclude clearly irrelevant studies. In cases where eligibility could not be determined from the abstract alone, the full-text articles were retrieved and reviewed for a more comprehensive assessment. The reference lists of included articles were also screened manually, although this did not yield any additional eligible studies.

All steps in the screening, study selection, data extraction and risk of bias assessment processes were conducted independently by two reviewers, with discrepancies resolved by discussion or consultation with a third reviewer.

### Data collection

Data were extracted using a standardized form and included: first author, publication year, country, study design, sample size, participant demographics (age, sex), clinical features (disease duration, ALSFRS-r scores, site of onset, FVC), NLR values, odds ratios (ORs) for disease progression and hazard ratios (HRs) for survival. In cases of missing data, corresponding authors were contacted; studies with unresolved missing data were excluded from the quantitative synthesis.^20,21,33,34^

### Data synthesis and meta-analysis

For studies reporting medians and interquartile ranges, values were converted to means and standard deviations using validated statistical methods.^35,36^

Heterogeneity across studies was assessed using the *I*^2^ statistic and Cochran’s Q test, with *I*^2^ values > 50% and *P*-values < 0.05 considered indicative of substantial heterogeneity.^37,38^ Publication bias was evaluated using funnel plots and Egger’s test, though interpretation was limited by the small number of studies (<10 per outcome).^39^ Certainty of evidence was assessed using the Grading of Recommendations Assessment, Development and Evaluation (GRADE) approach, considering risk of bias, inconsistency, indirectness, imprecision and publication bias.^40^

Given the typically skewed distribution of NLR, values were log-transformed prior to pooling. Pooled analyses were conducted separately for ALS patients and control groups. Standardized mean differences with 95% confidence intervals (CIs) were calculated for group comparisons, using a random-effects model with restricted maximum likelihood (REML) estimation. ORs and HRs were also pooled using random-effects models to evaluate associations with disease progression and survival, respectively. All statistical analyses were conducted in R version 4.5.0.

Meta-regression analyses were conducted to investigate potential sources of heterogeneity in HRs across studies. LogHRs and their standard errors were computed from reported CIs. Sample size and mean age were used as moderators. Model diagnostics, including tests for residual normality and multicollinearity, were assessed, though findings were interpreted with caution due to the limited number of studies.

### Quality appraisal

The methodological quality of the included studies was assessed using the Joanna Briggs Institute critical appraisal tools for diagnostic test accuracy studies and case reports.^41^

## Results

**Figure 1:**
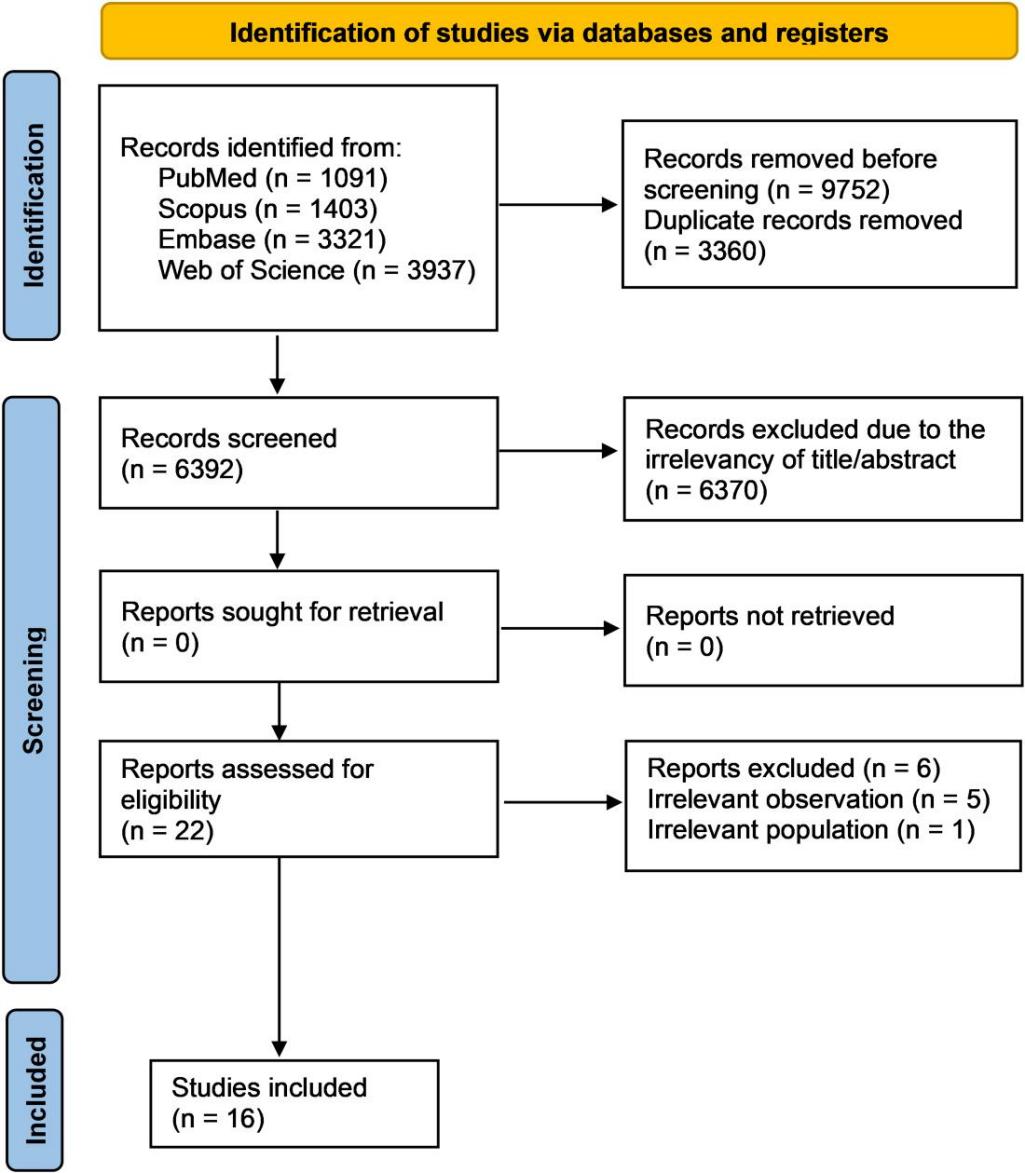
**Study selection flowchart.** PRISMA flow diagram illustrating the study selection process.

A total of 9752 articles were initially identified, of which 16 were ultimately included.^17-22,24-26,29,30,33,34,42-44^ The study selection process is detailed in the PRISMA flow diagram (Fig. 1).

Quality assessment of the included studies is presented in Table 1.

**Table 1:** Quality assessment of included studies using the JBI critical appraisal checklists

Diagnostic test accuracy studies										
Study	Q1	Q2	Q3	Q4	Q5	Q6	Q7	Q8	Q9	Q10
**Wang, 2025** ^33^	Yes	Yes	Yes	Unclear	N/A	Yes	Yes	Yes	Yes	No
**Hong, 2025** ^42^	Yes	No	Yes	Unclear	N/A	Yes	Yes	Yes	Yes	No
**Nona, 2024** ^44^	No	Yes	Yes	Unclear	No	Yes	Yes	Yes	Yes	No
**Femiano, 2024** ^43^	Yes	No	Yes	Unclear	N/A	Yes	Yes	Yes	Yes	Yes
**Liu, 2024** ^8^	Yes	No	Yes	Unclear	N/A	Yes	Yes	Yes	Yes	Yes
**Cao, 2023** ^17^	Yes	Yes	Yes	Yes	N/A	Yes	Yes	Yes	Yes	Yes
**Cotet, 2023** ^19^	Yes	Yes	Yes	Unclear	No	Yes	Yes	Yes	Yes	No
**Grassano, 2023** ^22^	Yes	Yes	Yes	Unclear	N/A	Yes	Yes	Yes	Yes	No
**Leone, 2022** ^25^	Yes	No	Yes	Unclear	No	Yes	Yes	Yes	Yes	No
**Wei, 2022** ^30^	Yes	Yes	Yes	Unclear	No	Yes	Yes	Yes	Yes	No
**Cui, 2022** ^20^	Yes	Yes	Yes	Unclear	N/A	Yes	Yes	Yes	Yes	No
**Santos, 2022** ^29^	Yes	Yes	Yes	Unclear	N/A	Yes	Yes	Yes	Yes	Yes
**Li, 2022** ^26^	Yes	No	Yes	Unclear	N/A	Yes	Yes	Yes	Yes	Yes
**Choi, 2020** ^18^	Yes	Yes	Yes	Unclear	N/A	Yes	Yes	Yes	Yes	Yes
**Keizman, 2009** ^24^	Yes	No	Yes	Unclear	N/A	Yes	Yes	Yes	Yes	No
Q1. Was a consecutive or random sample of patients enrolled? Q2. Was a case control design avoided? Q3. Did the study avoid inappropriate exclusions? Q4. Were the index test results interpreted without knowledge of the results of the reference standard? Q5. If a threshold was used, was it pre-specified? Q6. Is the reference standard likely to correctly classify the target condition? Q7. Were the reference standard results interpreted without knowledge of the results of the index test? Q8. Was there an appropriate interval between index test and reference standard? Q9. Did all patients receive the same reference standard? Q10. Were all patients included in the analysis?										
Case reports										
**Study**	**Q1**	**Q2**	**Q3**	**Q4**	**Q5**	**Q6**	**Q7**	**Q8**		
De Jesus-Morales, 2024^21^	Yes	No	Yes	Yes	N/A	N/A	N/A	Yes		
Q1. Were patient’s demographic characteristics clearly described? Q2. Was the patient’s history clearly described and presented as a timeline? Q3. Was the current clinical condition of the patient on presentation clearly described? Q4. Were diagnostic tests or methods and the results clearly described? Q5. Was the intervention(s) or treatment procedure(s) clearly described? Q6. Was the post-intervention clinical condition clearly described? Q7. Were adverse events (harms) or unanticipated events identified and described? Q8. Does the case report provide takeaway lessons?										

N/A, not applicable; Q, question.

A total of 357 044 participants were included across the studies, originating from 12 countries/territories: China, Italy, Israel, Puerto Rico, the United Kingdom, France, Sweden, Brazil, South Korea, Moldova, Romania and the USA. They comprised 8710 ALS patients (mean age: 60.25 years; 59.1% male) and 348 334 controls (mean age: 57.75 years; 47.6% male). Control groups varied across studies, including healthy controls,^17,24,34,42^ patients without degenerative or inflammatory diseases,^43^ ALS patients but without autoimmune diseases,^26^ as well as validation cohorts consisting of ALS patients.^25^ A detailed overview of the included studies and participant characteristics is presented in Supplementary Table 1.

### Diagnostic value of NLR

NLR was found to be elevated in ALS patients compared with healthy controls.^17,24,42^ However, no significant differences were observed when ALS patients were compared with patients without degenerative or inflammatory diseases.^43^ Additionally, NLR levels did not differ between ALS patients with or without autoimmune diseases, nor between those receiving or not receiving immunotherapy, even after adjusting for sex, age at onset and disease duration.^26^ Cao *et al*. found that higher NLR was consistently associated with an increased risk of incident ALS, particularly in participants younger than 65 years and those with BMI ≥ 25, independent of demographic, lifestyle and sociological factors.^17^ Keizman *et al*.^24^ reported that NLR independently distinguished ALS patients from controls, with each unit increase in NLR associated with 1.96-fold higher odds of ALS.

**Figure 2:**
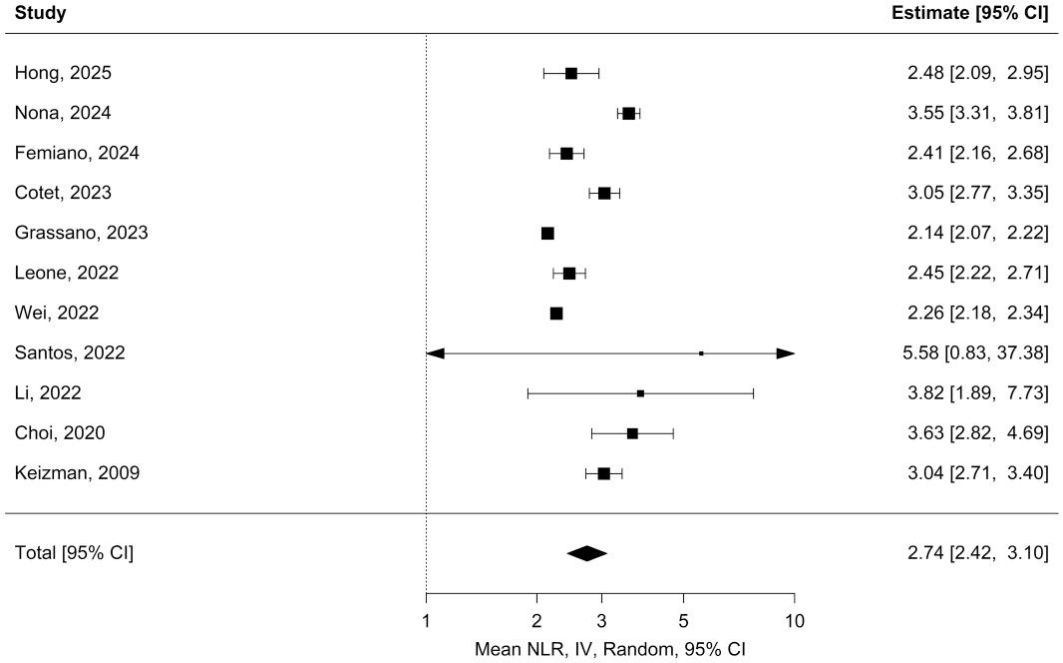
**Pooled mean NLR in ALS patients.** Forest plot of pooled mean NLR in ALS patients. Eleven studies (*N* = 4390) estimated a pooled mean NLR of 2.74 [95% CI (2.42, 3.10); *P* < 0.001], calculated using a random-effects meta-analysis model (restricted maximum likelihood).

**Figure 3:**
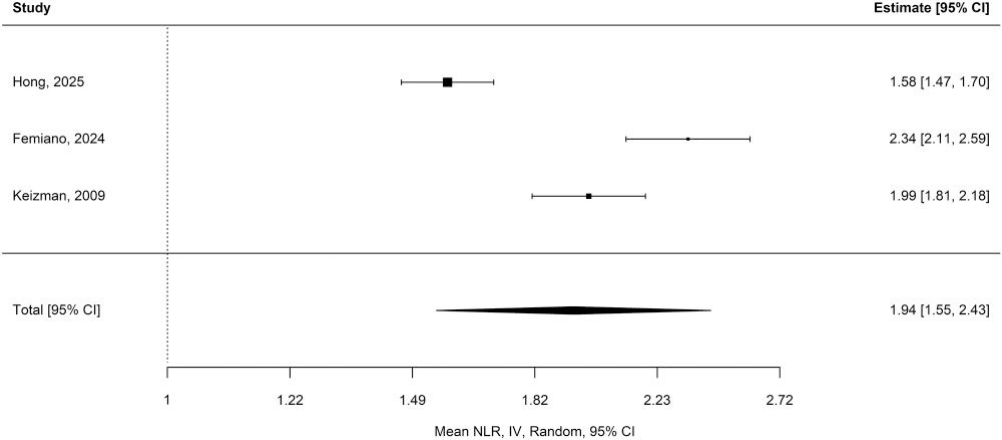
**Pooled mean NLR in control cohorts.** Forest plot of pooled mean NLR in control cohorts. Three studies (*N* = 227) estimated a pooled mean NLR of 1.94 [95% CI (1.55, 2.43); *P* < 0.001], calculated using a random-effects meta-analysis (restricted maximum likelihood).

The meta-analysis of 11 studies (*N* = 4390) estimated a pooled mean NLR of 2.74 [95% CI (2.42, 3.10); *P* < 0.001] in the ALS cohort, with high heterogeneity (*I*^2^ = 95.37%; Q_(10)_ = 225.98, *P* < 0.001) (Fig. 2).^18,19,22,24-26,29,30,42-44^ The pooled mean NLR from three studies (*N* = 227) in the control cohort was 1.94 [95% CI (1.55, 2.43); *P* < 0.001], with high heterogeneity (*I*^2^ = 94.71%; Q(_2_) = 39.71, *P* < 0.001) (Fig. 3).^24,42,43^

**Figure 4:**
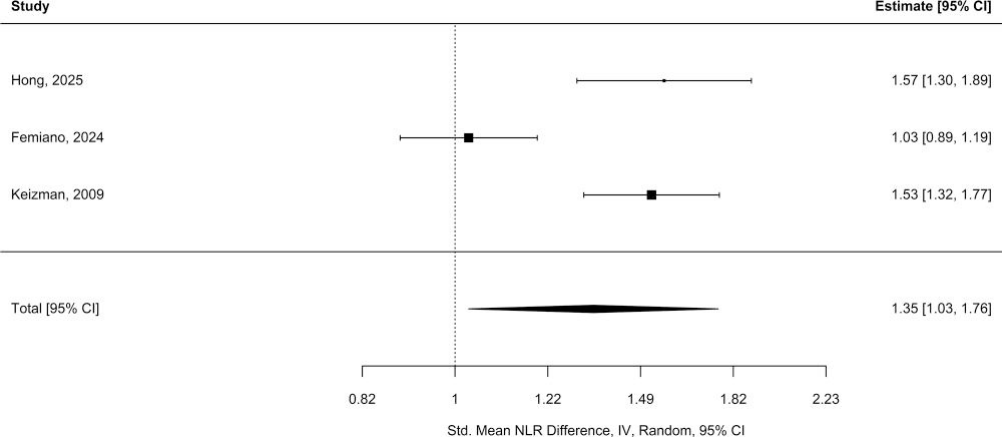
**Comparison of NLR between ALS and controls.** Forest plot comparing NLR between ALS patients and controls. Three studies (*N* = 208 ALS; *N* = 227 controls) showed a significantly higher NLR in ALS patients, with a pooled standardized mean difference of 1.35 [95% CI (1.03, 1.76); *P* = 0.03], calculated using a random-effects meta-analysis (restricted maximum likelihood).

Three studies (*N* = 208 ALS patients; *N* = 227 controls) were pooled to compare NLR (Fig. 4).^24,42,43^ A significant difference between the groups [1.35; 95% CI (1.03, 1.76); *P* = 0.03], indicated that on average, patients with ALS had a 35% higher NLR compared with controls. Heterogeneity was substantial (*I*^2^ = 88.28%; Q_(2)_ = 17.93, *P* < 0.001). Egger’s regression test for funnel plot asymmetry indicated no significant small-study effects (z = 0.6, *P* = 0.55). The estimated intercept, representing the effect size in a hypothetical infinitely large study, was −0.52 [95% CI (−3.25, 2.20)], suggesting minimal risk of publication bias. Based on the GRADE framework, the certainty of evidence for an elevated NLR in ALS compared with controls is *low*, primarily due to the observational study design and substantial heterogeneity among studies.

### Prognostic value of NLR

Across the included studies, elevated NLR was consistently associated with lower ALSFRS-r scores,^19,22,33,42^ higher progression rates,^19,22,33,42^ decreased survival time and increased mortality rate^19,20,25,30^ and in some studies, decreased FVC^18,19,22^ (Table 2). Longitudinal analyses highlighted a progressive increase in NLR over time.^18,19,21^ Moreover, temporal changes in NLR were highlighted in a case report by De Jesus-Morales, which reported a sharp rise in NLR at the point of symptom onset, suggesting its potential role as an early biomarker of disease activity.^21^ Notably, one study reported that NLR was only associated with ALSFRS-r and FVC at diagnosis, with no significant associations observed at 3, 6 or 12 months post-diagnosis.^19^

**Table 2:** Summary of key clinical and prognostic findings relating ‘higher’ NLR to ALS outcomes across included studies

Study	Outcomes	
	Significant	Non-significant
**Wang, 2025^33^**	Lower ALSFRS-r scores, higher ΔFS, increasing compound muscle action potential relative scores, more severe lower motor neuron axonal dysfunction (particularly in female patients and those younger than 50 years)	N/A
**Hong, 2025^42^**	Lower ALSFRS-r scores, higher disease progression rate after diagnosis, shorter survival and increased mortality risk	N/A
**Nona, 2024^44^**	Male sex, older onset age, classical ALS and shorter survival from symptom onset	Onset site, family history of ALS, FVC, BMI and ALSFRS-r scores
**Femiano, 2024^43^**	PC1^a^ and medium/high disease progression rate CSF cytokines (IL-6, G-CSF, MIP-1a and MIP-1b, although not significant after controlling for multiple comparisons)	N/A
**Liu, 2024^34^**	Increased Prevotella-1 and Lachnospiraceae_NK4A136_group and decreased Streptococcus in nasal microbiota	N/A
**De Jesus-Morales, 2024^21^**	Symptom onset (sharp increase in NLR)	N/A
**Cao, 2023^17^**	Higher BMI (> 25)	Sex
**Cotet, 2023^19^**	Decreased FVC, decreased ALSFRS-r scores, lower BMI, shorter disease duration and increased probability of decreased survival FVC at diagnosis ALSFRS-r scores at diagnosis and after 3 months Increased disease progression rate and shorter survival at inclusion, and 6 and 12 months after diagnosis	Sex, age at diagnosis and onset site FVC at 3, 6 and 12 months after diagnosis ALSFRS-r scores at 6 and 12 months after diagnosis
**Grassano, 2023^22^**	Older age, lower ALSFRS-r scores at diagnosis, increased ΔFS rate, decreased FVC, disease stage (65–75 years) and decreased survival time	Sex, diagnostic delay, onset site, active smoking, bulbar symptoms and cognitive impairment
**Leone, 2022^25^**	Older age (at onset, diagnosis and recruitment), decreased ALSFRS-r scores, increased ΔFS rate, mortality rate and survival time	Sex, disease duration, onset site, Escorial ALS, FVC, BMI and use of riluzole
**Wei, 2022^30^**	Older age, male sex, onset age, early disease stage, classical phenotype, onset region (bulbar), decreased ALSFRS-r scores, higher rate of disease progression, decreased survival and increased mortality risk	BMI, diagnostic duration, diagnostic delay
**Cui, 2022^20^**	Higher rate of disease progression and mortality	N/A
**Santos, 2022^29^**	Higher platelet-to-lymphocyte ratio in both initial and advanced stages	Disease stage (initial/advanced)
**Li, 2022^26^**	N/A	Autoimmune diseases, immunotherapy and survival time
**Choi, 2020^18^**	Older onset age, decreased FVC, shorter survival time and higher neutrophil-to-monocyte ratio	Sex, BMI, months from onset to diagnosis, onset site, disease duration, ALSFRS-r scores, progression rate, use of riluzole and the levels of total cholesterol, albumin, creatinine, uric acid and CRP
**Keizman, 2009^24^**	N/A	ALSFRS-r scores and disease progression

ΔFS, Delta ALS-FRS-r score; ALSFRS-r, Amyotrophic Lateral Sclerosis Functional Rating Scale-Revised; BMI, body mass index; CRP, C-reactive protein; FVC, forced vital capacity; G-CSF, granulocyte colony stimulating factor; IL, interleukin; MIP,macrophage inflammatory protein; N/A, not applicable; NLR, neutrophil-to-lymphocyte ratio; PC1, principal component 1 (main component in PCA analysis of cytokines).

^a^PC1: particularly IL-9, IL-4, GCSF, IL-7, IL-17, IL-13, IL-6, IL-1β, TNF, IL-2, associated with ALS diagnosis.

**Figure 5:**
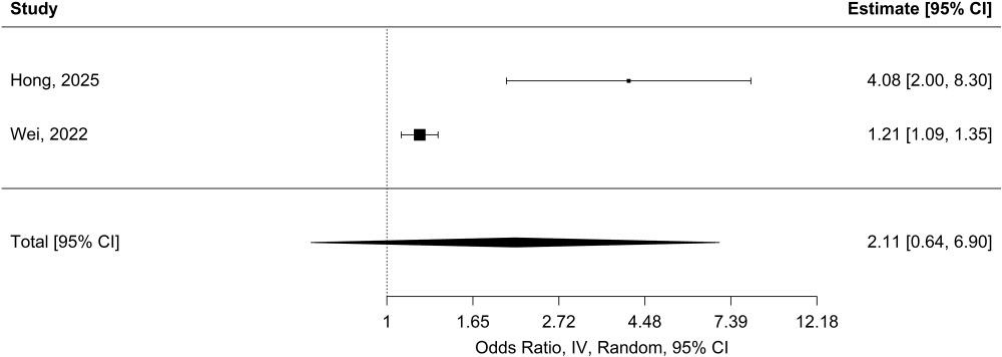
**Pooled ORs for NLR and ALS progression.** Forest plot of pooled odds ratios for NLR and ALS progression (two studies). Pooled ORs were estimated using a random-effects model (estimator: restricted maximum likelihood), with 95% CI and *z*-test *P*-values. Elevated NLR was associated with OR = 2.11 [95% CI (0.64, 6.90); *P* = 0.22].

Across studies, higher NLR was generally associated with faster ALS progression,^19-22,24,25,30,33,42,43^ although a few reports found no statistically significant association.^18,24,29^ More specifically, Hong *et al*.^42^ found that higher NLR was associated with a more than fourfold increased risk of rapid progression [OR = 4.54; 95% CI (1.90, 10.82); *P* = 0.001], after adjustment for sex, age at onset, diagnostic delay, site of onset, BMI at diagnosis and riluzole use. Wei *et al*.^30^ reported a statistically significant but more modest association [OR = 1.21; 95% CI (1.09, 1.35); *P* < 0.001], adjusted for age at onset, sex, onset region, BMI, HbA1c and albumin. A random-effects meta-analysis using REML was conducted across two studies. The pooled effect on the log scale was an OR of 2.11 [95% CI (0.64, 6.90); *P* = 0.22], indicating a nonsignificant trend towards increased odds (Fig. 5), with substantial heterogeneity (*I*^2^ = 90.9%; Q_(1)_ = 10.98; *P* = 0.0009).

Survival analyses across included studies consistently demonstrated that elevated NLR predicts worse outcomes in ALS. Choi *et al*.^18^ reported that participants in the highest NLR tertile (mean 2.91 ± 0.85) had higher mortality than those in the lowest tertile (mean 1.14 ± 0.26), both unadjusted [HR = 1.79; 95% CI (1.15, 2.79); *P* = 0.009] and adjusted for clinical covariates [HR = 1.60; 95% CI (1.01, 2.51); *P* = 0.041], whereas the middle tertile showed no significant difference from the lowest. Additionally, Cui *et al*.^20^ reported that each standard deviation increase in NLR corresponded to a substantially higher risk of death [HR = 1.31; 95% CI (1.13, 1.52)]).

**Figure 6:**
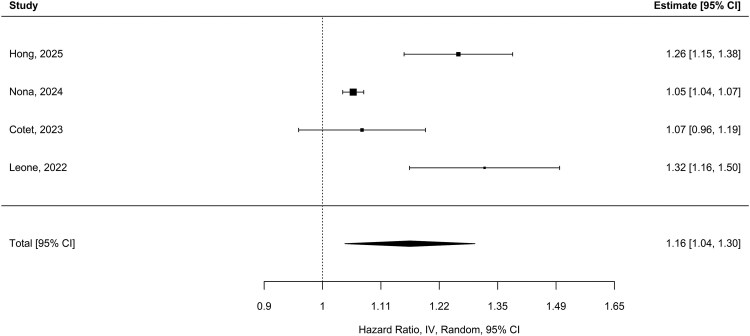
**Pooled univariate HRs for NLR and survival.** Forest plot of pooled univariate hazard ratios for NLR and survival in ALS (four studies). Pooled HRs were estimated using a random-effects model (estimator: restricted maximum likelihood), with 95% CI and *z*-test *P*-values. Higher NLR was associated with an increased risk of death [HR = 1.16; 95% CI (1.04, 1.29); *P* = 0.009].

The pooled univariate HR of four studies was 1.16 [95% CI (1.04, 1.29); *P* = 0.009], with high heterogeneity (*I*^2^ = 93.8%; Q_(3)_ = 24.82; *P* < 0.001) (Fig. 6).^19,25,42,44^ Egger’s test indicated evidence of small-study effects or potential publication bias (z = 3.8; *P* = 0.0002). After adjusting for age and sample size, residual heterogeneity decreased to *I*^2^ = 40.3%, with these moderators accounting for 78.8% of the between-study variability. Age was not significantly associated with HR [HR per year = 0.99; 95% CI (0.97, 1.01); *P* = 0.32], whereas sample size was significantly associated [HR per unit increase = 0.918; 95% CI (0.86, 0.99); *P* = 0.008], indicating that larger studies tended to report slightly lower HR estimates. According to GRADE, the association between elevated NLR and increased hazard is of low-to-moderate certainty, reflecting a statistically significant pooled HR but limited by high heterogeneity, small-study effects and the small number of included studies.

**Figure 7:**
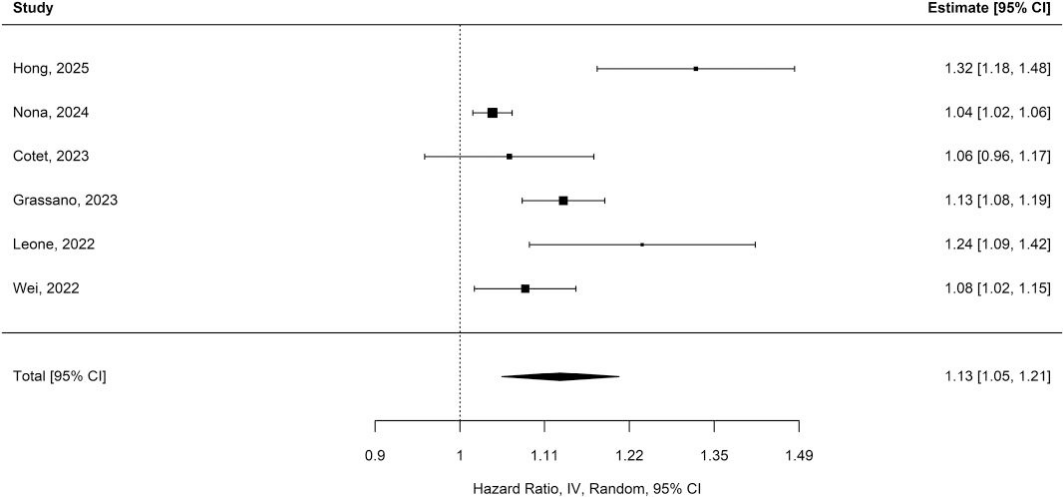
**Pooled multivariate HRs for NLR and survival.** Forest plot of pooled multivariate hazard ratios for NLR and survival in ALS (six studies). Pooled HRs were estimated using a random-effects model (estimator: restricted maximum likelihood), with 95% CI and *z*-test *P*-values. After adjustment for clinical covariates, elevated NLR remained an independent predictor of poorer survival [HR = 1.13; 95% CI (1.06, 1.21); *P* = 0.004].

The multivariable HR of six studies was 1.13 [95% CI (1.06, 1.21); *P* < 0.001], with high heterogeneity (*I*^2^ = 86.5%; Q_(5)_ = 28.38; *P* < 0.001) (Fig. 7).^19,22,25,30,42,44^ Egger’s test indicated evidence of small-study effects or potential publication bias (*z* = 2.44; *P* = 0.015). After adjusting for age and sample size, residual heterogeneity remained high (*I*^2^ = 82.2%), with these moderators accounting for 30.4% of the between-study variability. Age was not significantly associated with HR [HR per year = 1.002; 95% CI (0.99, 1.02); *P* = 0.78], whereas log-transformed sample size was significantly associated [HR per unit increase = 0.94; 95% CI (0.88, 0.99); *P* = 0.043], indicating that larger studies tended to report slightly lower HR estimates. According to GRADE, the association between elevated NLR and increased hazard is of low-to-moderate certainty, reflecting a statistically significant pooled HR but limited by high heterogeneity, small-study effects and residual confounding.

### Other outcomes

Correlations of higher NLR with various clinical and demographic factors are summarized in Table 2. Some studies reported that higher NLR values were more frequently observed in patients with older age at onset, male sex and the classical ALS phenotype. However, these associations were not consistent across all studies. BMI, disease onset site and diagnostic delay similarly showed no consistent associations across studies, nor with other parameters such as riluzole use, disease duration or bulbar symptoms in several cohorts. Notably, one study observed increased NLR in ALS patients over 75 years old with overlapping frontotemporal dementia.^22^

Beyond demographic and clinical correlates, NLR was also explored in relation to inflammatory and immunological markers. One study reported that elevated NLR levels were associated with the combined activity of CSF cytokines (IL-6, G-CSF, MIP-1a and MIP-1b), although this association was not significant after controlling for multiple comparisons.^43^ Moreover, a study of the nasal microbiota (the alpha and beta diversity and relative abundance of bacterial taxa) in ALS patients found reduced alpha diversity compared with healthy controls. Also, NLR was positively correlated with Prevotella-1 and Lachnospiraceae_NK4A136_group, and negatively correlated with Streptococcus.^34^ Furthermore, one study reported that lower motor neuron axonal dysfunction mediated approximately 53.3% of the effect of NLR on disease progression, highlighting a potential mechanistic link between systemic inflammation and neurodegeneration in ALS.^33^

## Discussion

This systematic review and meta-analysis synthesized evidence from 16 studies investigating NLR in ALS. Pooled analyses revealed that NLR levels were significantly higher in ALS patients compared with healthy controls. Elevated NLR was consistently associated with faster disease progression, reduced survival and increased mortality. However, effect sizes varied considerably across studies, with substantial heterogeneity detected. Additional findings suggested potential demographic and clinical moderators of NLR, such as age at onset and phenotype, although these associations were not consistent.

Elevated NLR values are consistently associated with worse outcomes in ALS, including faster progression, shorter survival and higher mortality. However, whether NLR contributes directly to pathogenesis via neutrophil-mediated damage or merely reflects underlying inflammation remains uncertain. Further research is needed to clarify NLR’s role in ALS and its potential as a diagnostic and prognostic biomarker, particularly in relation to systemic inflammation, immune dysregulation and microbiota alterations. NLR increases with age in both sexes,^15,18^ aligning with evidence that the brain becomes progressively more proinflammatory over time.^45^ Also, studies in Alzheimer’s disease and the general population similarly suggest that systemic inflammation emerges later in disease progression and ageing.^46^ Moreover, a case report by De Jesus *et al*. described a rise in NLR during early ALS symptoms, which normalized briefly after edaravone treatment, alongside slight improvements in pulmonary function, highlighting the dynamic nature of inflammatory responses to therapy.^21^ In line with this concept, a recent report on low-dose IL-2 therapy suggests that modulating age-related increases in inflammation and restoring regulatory T cell (T-reg) function may offer an additional therapeutic avenue worth exploring in the context of ALS immune dysregulation.^47^ However, recent evidence indicates that elevated NLR may occur even before motor symptoms, supporting its potential as an early biomarker for ALS.^17^

The association between NLR and ALS progression is complex and multifaceted. Dysregulated inflammatory pathways, marked by increased pro-inflammatory cytokines and neutrophil infiltration into the central nervous system, are thought to exacerbate neurodegeneration in ALS. Peripheral myeloid cells are functionally altered in ALS patients.^48^ Neutrophils can accumulate in the spinal cord,^28,49^ causing damage via oxidative stress^27^ and altering microglial states, thus worsening motor neuron degeneration.^25^ Pathological changes in ALS brain and spinal tissue include deposits of fibrinogen, thrombin, IgG, hemosiderin, pericyte and endothelial degeneration, disrupted tight junctions, basement membrane changes and enlarged perivascular spaces, indicating blood-brain and blood-spinal cord barrier disruption.^50,51^ Elevated CSF albumin and IgG levels further support barrier impairment.^52^ In skeletal muscle, neutrophil infiltration interacts with mast cells, promoting neuromuscular junction denervation and muscle atrophy via proteases and reactive oxygen species, contributing to disease severity.^53,54^ Increased blood neutrophils may reflect bone marrow production rather than reduced CNS recruitment. Neuroinflammation is also driven by microglial activation, while low T lymphocyte counts correlate with rapid disease progression.^30,55^ Systemic inflammation, possibly mediated by IL-6, may exacerbate blood-brain barrier breakdown and neurodegeneration vulnerability.^50,56^ Similar inflammatory processes and blood-brain barrier disruption are linked to faster cognitive decline in dementia.^57^ Lymphocytes play a stage-dependent role in ALS. Higher lymphocyte counts are linked to better survival.^58^ T-regs offer neuroprotection through anti-inflammatory cytokines and modulation of microglia, but their function is impaired in ALS.^59^ In contrast, CD8+ T cells may promote motor neuron death.^60^ Additionally, growing evidence shows that immunosenescence and metabolic alterations in lymphocytes also contribute to immune dysregulation. Proteomic profiling has identified enrichment of senescence- and metabolism-related pathways in fast-progressing patients,^61^ and senescent-like lymphocytes have been shown to correlate with disease progression and survival.^62^ T-regs secrete anti-inflammatory cytokines (IL-4, IL-10, IL-13) and neurotrophic factors, transform Th1 to Th2 response and attenuate toxic microglial responses directly differentiating macrophages from M1 to M2 state, and directly inhibit *IL-2* mRNA transcription.^63^ Increased CNS infiltration of T-regs and Th2 cells in slow-progressing patients suggests a protective role,^64^ supported by animal models showing T-regs delay disease progression.^30,65^ Additionally, animal models have suggested that early lymphocyte infiltration may be harmful, while later infiltration appears beneficial.^66^

Emerging evidence suggests a potential link between systemic inflammation and microbiota dysbiosis in ALS. Dysbiosis of the nasal and gut microbiota in ALS patients may trigger inflammatory responses and impact immune function, highlighting the interconnectedness of the gut-brain axis in ALS pathogenesis. ALS patients with lower NLR levels show greater gut microbial diversity,^67^ which may influence disease progression, as supported by both animal^68,69^ and human studies.^70^ The gut-immune interface plays a key role in immune regulation, with the intestinal barrier hosting complex interactions between microbiota, epithelial cells and immune components. Similar to the gut, ALS patients also exhibit altered nasal microbiota, potentially contributing to immune dysregulation and inflammation.^34,71,72^ Nasal dysbiosis has been associated with other neurological conditions, including Alzheimer’s disease and stroke.^73,74^ Specific bacteria, such as Klebsiella and Sphingomonas, may promote inflammation or signal neural damage.^75,76^ Notably, Prevotella-9 correlates with better pulmonary function in ALS,^77^ though it is often depleted in the gut.^78^ Altered glycan synthesis and metabolic activity have also been reported in ALS patients, potentially disrupting energy homeostasis and immune regulation.^79^ Increased glycolysis has been linked to motor neuron degeneration, while gut microbiota can modulate microglial activity, key players in neuroinflammation and neuroprotection in ALS.^80,81^ However, microglial phenotype and function are responsive to external stimuli, including signals from commensal microbiota. Taken together, these findings highlight the central role of the microbiome-immune axis in shaping systemic inflammation in ALS. Building on this, dietary patterns and ethnicity, both major determinants of gut microbiome composition, also influence systemic inflammatory tone, as reflected by NLR.^82-86^ Population studies show that unhealthy diets and adiposity are associated with higher NLR, whereas plant-rich dietary patterns correlate with lower NLR and distinct microbiome profiles. Baseline leukocyte counts and NLR additionally vary across ethnic groups, underscoring population-level differences in inflammatory status. Because diet and ethnicity strongly shape microbial diversity, these factors likely interact with microbiome alterations observed in ALS. In this context, the co-occurrence of elevated NLR and gut dysbiosis supports a microbially driven pro-inflammatory state, suggesting that dietary intake and ethnic background may contribute to individual variability in systemic inflammation in ALS.

Despite a rigorous methodology, this review has several limitations that warrant caution. The prognostic value of NLR must be considered within established ALS multivariate frameworks that incorporate age of onset, diagnostic category, phenotype, site of onset, BMI, ALSFRS-r and its slope, FVC, respiratory status and use of riluzole. NLR is also affected by comorbidities and medications such as corticosteroids, which further limits its reliability as a diagnostic or prognostic biomarker. However, the included studies reported and adjusted for these variables inconsistently, contributing to heterogeneity, limiting confounder adjustment and meta-regression and restricting subgroup analyses, despite evidence that inflammatory profiles differ across clinical and demographic groups. More recent prognostic models that integrate clinical factors with NfL^12^ and ALSFRS-r slope^87^ demonstrate markedly improved accuracy, yet none of the NLR studies incorporated NfL and few included ALSFRS-r slope, limiting evaluation of the independent contribution of NLR. Several comparisons were additionally based on small sample sizes, further reducing statistical power.

Variability in disease duration, clinical status and inconsistently timed single-point NLR measurements, along with the inclusion of both incident and prevalent cases, complicates interpretation, especially since potential fluctuations in NLR during ALS progression have not been validated longitudinally. Most studies also categorized NLR rather than modelling it as a continuous variable, reducing the ability to assess dose-response relationships. Substantial heterogeneity across pooled analyses suggests unmeasured effect modifiers that limit precision and generalizability, which is further constrained by the predominance of single-centre or region-specific cohorts. Mechanistic and biomarker data were largely absent, and the role of NLR in ALS pathophysiology therefore remains associative rather than causal.

Future research should employ longitudinal designs, standardized NLR assessment, integration with established molecular biomarkers (ideally within multi-biomarker prognostic frameworks), and subgroup-specific analyses across diverse ALS populations. Although NLR may ultimately enhance prognostication and support individualized therapeutic strategies aimed at modulating inflammation in ALS, until such evidence is available, it should be interpreted cautiously and within the broader landscape of diagnostic and prognostic indicators.

## Conclusion

This systematic review and meta-analysis demonstrates that elevated NLR is associated with faster disease progression, reduced survival and increased mortality in ALS, supporting its potential utility as a readily accessible, cost-effective and non-invasive biomarker reflecting systemic inflammation and disease activity in ALS. However, substantial heterogeneity across studies, inconsistent adjustment for key clinical predictors, variable timing of NLR measurement and limited subgroup or biomarker integration underscore the need for more rigorous evaluation. Future prospective and longitudinal studies incorporating standardized NLR assessment, established clinical predictors and multi-biomarker frameworks are essential to determine whether NLR offers independent and clinically meaningful prognostic value in ALS.

## Supplementary Material

fcag132_Supplementary_Data

## Data Availability

Data sharing is not applicable to this article as no datasets or codes were generated or analysed during the current study.
