# Supplementary material for: Neutrophil-to-lymphocyte ratio in amyotrophic lateral sclerosis: a systematic review and meta-analysis
Source: Brain Commun. 2026 Apr 11;8(2):fcag132. doi: 10.1093/braincomms/fcag132 (PMC13111489; doi:10.1093/braincomms/fcag132)
Supplement: fcag132_Supplementary_Data [file fcag132_supplementary_data.docx]

**Supplementary Material 1**

1. “Amyotrophic Lateral Sclerosis”[MeSH Terms]
2. “Amyotrophic Lateral Sclerosis”[Title/abstract]
3. ALS[Title/Abstract]
4. “Gehrig Disease”[Title/abstract]
5. “Gehrig’s Disease”[Title/abstract]
6. “Lytico bodig Disease”[Title/abstract]
7. “Guam Disease”[Title/abstract]
8. “Charcot Disease”[Title/abstract]
9. “Charcot's Disease”[Title/abstract]
10. “Motor Neuron Disease”[MeSH Terms]
11. “Motor Neuron Disease”[Title/abstract]
12. MND[Title/abstract]
13. #1 OR #2 OR #3 OR #4 OR #5 OR #6 OR #7 OR #8 OR #9 OR #10 OR #11 OR #12
14. Neutrophils[MeSH Terms]
15. Neutrophil*[Title/Abstract]
16. Lymphocytes[MeSH Terms]
17. Lymphocyt*[Title/Abstract]
18. “Neutrophil to Lymphocyte Ratio”[Title/Abstract]
19. “Neutrophil Lymphocyte Ratio”[Title/Abstract]
20. “Neutrophil-to-Lymphocyte Ratio”[Title/Abstract]
21. “Neutrophil-Lymphocyte Ratio”[Title/Abstract]
22. NLR[Title/Abstract]
23. #14 OR #15 OR #16 OR #17 OR #18 OR #19 OR #20 OR #21 OR #22
24. #13 AND 23

- **PubMed**

((“Amyotrophic Lateral Sclerosis”[MeSH Terms] OR “Amyotrophic Lateral Sclerosis”[Title/abstract] OR ALS[Title/Abstract] OR “Gehrig Disease”[Title/abstract] OR “Gehrig’s Disease”[Title/abstract] OR “Lytico bodig Disease”[Title/abstract] OR “Guam Disease”[Title/abstract] OR “Charcot Disease”[Title/abstract] OR “Charcot's Disease”[Title/abstract] OR “Motor Neuron Disease”[MeSH Terms] OR “Motor Neuron Disease”[Title/abstract] OR MND[Title/abstract]) AND (Neutrophils[MeSH Terms] OR Neutrophil*[Title/Abstract] OR Lymphocytes[MeSH Terms] OR Lymphocyt*[Title/Abstract] OR “Neutrophil to Lymphocyte Ratio”[Title/Abstract] OR “Neutrophil Lymphocyte Ratio”[Title/Abstract] OR “Neutrophil-to-Lymphocyte Ratio”[Title/Abstract] OR “Neutrophil-Lymphocyte Ratio”[Title/Abstract] OR NLR[Title/Abstract]))

- **WOS**

TS=(“Amyotrophic Lateral Sclerosis” OR “Amyotrophic Lateral Sclerosis” OR ALS OR “Gehrig Disease” OR “Gehrig’s Disease” OR “Lytico bodig Disease” OR “Guam Disease” OR “Charcot Disease” OR “Charcot's Disease” OR “Motor Neuron Disease” OR “Motor Neuron Disease” OR MND)

TS=(Neutrophils OR Neutrophil* OR Lymphocytes OR Lymphocyt* OR “Neutrophil to Lymphocyte Ratio” OR “Neutrophil Lymphocyte Ratio” OR “Neutrophil-to-Lymphocyte Ratio” OR “Neutrophil-Lymphocyte Ratio” OR NLR)

- **Scopus**

TITLE-ABS((“Amyotrophic Lateral Sclerosis” OR ALS OR “Gehrig Disease” OR “Gehrig’s Disease” OR “Lytico bodig Disease” OR “Guam Disease” OR “Charcot Disease” OR “Charcot's Disease” OR “Motor Neuron Disease” OR MND) AND (Neutrophils OR Neutrophil* OR Lymphocytes OR Lymphocyt* OR “Neutrophil to Lymphocyte Ratio” OR “Neutrophil Lymphocyte Ratio” OR “Neutrophil-to-Lymphocyte Ratio” OR “Neutrophil-Lymphocyte Ratio” OR NLR))

- **Embase**

(('amyotrophic lateral sclerosis'/exp OR 'amyotrophic lateral sclerosis':ti,ab,kw OR 'als':ti,ab,kw OR 'gehrig disease':ti,ab,kw OR 'gehrig`s disease':ti,ab,kw OR 'lytico bodig disease':ti,ab,kw OR 'guam disease':ti,ab,kw OR 'charcot disease':ti,ab,kw OR 'charcot`s disease':ti,ab,kw OR 'motor neuron disease'/exp OR 'motor neuron disease':ti,ab,kw OR 'mnd':ti,ab,kw) AND ('neutrophil'/exp OR 'neutrophil*':ti,ab,kw OR 'lymphocyte'/exp OR 'lymphocyt*':ti,ab,kw OR 'neutrophil to lymphocyte ratio':ti,ab,kw OR 'neutrophil lymphocyte ratio':ti,ab,kw OR 'neutrophil-to-lymphocyte ratio':ti,ab,kw OR 'neutrophil-lymphocyte ratio':ti,ab,kw OR 'nlr':ti,ab,kw))

**Supplementary Material 2**

Supplementary Table 1. Detailed overview of the characteristics of included studies and participant demographics

| Study | Country | Study design | Diagnostic criteria | Sample size | Participants’ characteristics | |
| --- | --- | --- | --- | --- | --- | --- |
|  |  |  |  |  | **ALS patients** | **Controls** |
| Wang, 2025 ^1^ | China | Prospective cohort | El Escorial | 372 | Age = 53.3 ± 11.9  Sex(M/F) = 240/132  Disease duration = 248.24 ± 84.09  ALSFRS-r = 43.21 ± 3.16  Site of onsite (Bulbar/Spinal) = 69/282  NLR: NR | N/A |
| Hong, 2025 ^3^ | China | Retrospective cohort | El Escorial | 145 | (n = 72)  Age = 61.39 ± 9.86  Age at onset = 60.37 ± 10.02  Sex(M/F) = 42/30  Disease duration (m) = 32.86 ± 17.88  ALSFRS-r = 34.7 ± 22.7  Site of onsite (Bulbar/Spinal) = 17/55  Progression rate = 1.04 ± 0.7  NLR = 2.48 ± 1.85 | Healthy controls (n = 73)  Age = 59.07 ± 5.97  Sex(M/F) = 40/33  NLR = 1.58 ± 0.52 |
| Nona, 2024 ^4^ | Israel | Retrospective cohort | El Escorial | 822 | Age = 61.3 ± 11.9  Sex(M/F) = 493/329  FVC = 80.6 ± 23.8  ALSFRS-r = 35.7 ± 6.9  Survival (m) = 36 ± 2.97  NLR = 3.55 ± 3.63 | N/A |
| Femiano, 2024 ^5^ | Italy | Cross-sectional | El Escorial | 103 | (n = 56)  Age = 63.51 ± 12.55  Sex(M/F) = 37/19  Disease duration (m) = 8.85 ± 4.56  ALSFRS-r = 43.64 ± 2.28  NLR = 2.41 ± 0.99 | Controls without degenerative/inflammatory diseases** (n = 47)  Age = 58.81 ± 10.40  Sex(M/F) = 28/19  NLR = 2.34 ± 0.83 |
| Liu, 2024 ^6^ | China | Cross-sectional | El Escorial | 106 | (n = 66)  Age = 58 ± 10.61  Sex(M/F) = 37/29  ALSFRS-r = 42.94 ± 3.79  NLR: NR | Healthy controls (n = 40)  Age = 58.22 ± 9.99  Sex(M/F) = 23/17  NLR: NR |
| De Jesus-Morales, 2024 ^7^ | Puerto Rico | Case report | El Escorial | 1 | Age = 56  Sex = Male  NLR: NR | N/A |
| Cao, 2023 ^8^ | United Kingdom | Retrospective cohort | El Escorial | 345,000 | (n = 348)  Age = 61.58 ± 6.84  Sex(M/F) = 196/152  NLR = 2.32 ± 0.8 | Healthy controls (n = 344,652)  Age = 56.3 ± 10.38  Sex(M/F) = 163,694/180,958  NLR = 2.16 ± 0.8 |
| Cotet, 2023 ^9^ | France | Retrospective cohort | El Escorial | 359 | Age = 67.3 ± 11.5  Sex(F/M) = 175/183  Disease duration = 32.5 ± 27.6  NLR = 3.05 ± 2.79 |  |
| Grassano, 2023 ^10^ | Italy | Retrospective cohort | El Escorial | 1452 | Age = 68.83 ± 10.61  Sex(F/M) = 652/799  NLR = 2.14 ± 1.47 | N/A |
| Leone, 2022 ^11^ | Italy, Moldova, Romania | Retrospective cohort | El Escorial | 3,588 | (n = 146)  Age = 61.18 ± 10.9  Sex(F/M) = 88/58  Disease duration = 19.72 ± 18.40  FVC (> 80% / < 80%): 51/50  ALSFRS-r = 35.78 ± 8.26  Progression rate = 0.76 ± 0.868  NLR = 2.45 ± 1.51 | Validation cohort (ALS patients) (n = 3,442)  Age = 55.50 ± 11.32  Sex(M/F) = 2,140/1,302  Disease duration = 19.23 ± 12.28  NLR = 2.82 ± 1.37 |
| Wei, 2022 ^12^ | China | Retrospective cohort | El Escorial | 1,030 | Age = 56.96 ± 11.33  Sex(M/F) = 606/424  ​Progression rate = 0.84 ± 0.75  NLR = 2.26 ± 1.33 | N/A |
| Cui, 2022 ^13^ | Sweden | Retrospective cohort | El Escorial | 92 | (n = 92)  Age = 62 ± 12.05  Sex(M/F) = 43/49  NLR: NR | N/A |
| Santos, 2022 ^14^ | Brazil | Cross-sectional | El Escorial | 43 | Age range = 31-81  Sex(M/F) = 35/8  Stage of disease (Early/Advanced) = 29/14  NLR = 1.72 [0.25, 11.57] | N/A |
| Li, 2022 ^15^ | Sweden | Retrospective cohort | El Escorial | 136 | ALS patients + autoimmune diseases* (n = 26)  Age of onset = 55.27 ± 7.94  Sex(M/F) = 13/13  Disease duration = 13.82 ± 9.02  NLR = 2.07 ± 0.94 | ALS patients without autoimmune diseases (n = 110)  Age of onset = 49.08 ± 12.10  Sex(M/F) = 63/47  Disease duration = 11.0 (6.8, 18.3)  NLR = 1.92 ± 0.82 |
| Choi, 2020 ^16^ | South Korea | Retrospective cohort | El Escorial | 194 | Age = 61.16 ± 11.15  Sex(M/F) = 109/85  ALSFRS-r = 38.94 ± 6.11  FVC (> 80% / < 80%): 63/74  Progression rate = 0.69 ± 0.58 ​  Survival (m) = 33.17 ± 21.63  NLR = 1.29 [1.003, 1.66] | N/A |
| Keizman, 2009 ^17^ | Israel | Prospective cohort | El Escorial | 160 | (n = 80)  Age = 59 ± 19  Sex(M/F) = 49/31  NLR = 3.04 ± 1.57 | Healthy controls (n = 80)  Age = 58.6 ± 12.8  Sex(M/F) = 49/31  NLR = 1.99 ± 0.84 |
| *Asthma, Graves’s disease, Hashimoto, Rheumatoid arthritis, Sjogren syndrome, Primary biliary cirrhosis, Ulcerative colitis, Scleroderma, Myasthenia gravis, Vitiligo, Chronic urticaria  **Vascular Leukoencephalopathy, Metabolic and Hereditary Polyneuropathies, Normal Pressure Hydrocephalus, Functional Neurological Disorder, Migraine, Spondylotic Myelopathy, and Spastic Paraparesis.  ΔFS: Delta ALS-FRS-r Score; (m): (months); ALS: Amyotrophic Lateral Sclerosis; ALSFRS-R: Amyotrophic Lateral Sclerosis Functional Rating Scale-Revised; F: Female; FVC: Forced Vital Capacity; M: Male; m: Months; N/A: Not Applicable; NLR: Neutrophil-to-Lymphocyte Ratio; NR: Not Reported. | | | | | | |

1. Wang Z, Cao W, Chen L*, et al*. The role of the peripheral immune system in mediating axonal dysfunction in early-stage amyotrophic lateral sclerosis: an age- and sex-based analysis. *Neural Regen Res*. Mar 25 2025;doi:10.4103/nrr.Nrr-d-24-01081

2. Bensimon G, Leigh PN, Tree T*, et al*. Efficacy and safety of low-dose IL-2 as an add-on therapy to riluzole (MIROCALS): a phase 2b, double-blind, randomised, placebo-controlled trial. *Lancet*. May 24 2025;405(10492):1837-1850. doi:10.1016/s0140-6736(25)00262-4

3. Hong Y, Shi JQ, Feng S*, et al*. The systemic inflammation markers as potential predictors of disease progression and survival time in amyotrophic lateral sclerosis. *Front Neurosci*. 2025;19:1552949. doi:10.3389/fnins.2025.1552949

4. Nona RJ, Henderson RD, McCombe PA. Neutrophil-to-lymphocyte ratio at diagnosis as a biomarker for survival of amyotrophic lateral sclerosis. *Amyotroph Lateral Scler Frontotemporal Degener*. Aug 2024;25(5-6):452-464. doi:10.1080/21678421.2024.2351187

5. Femiano C, Bruno A, Gilio L*, et al*. Inflammatory signature in amyotrophic lateral sclerosis predicting disease progression. *Sci Rep*. Aug 27 2024;14(1):19796. doi:10.1038/s41598-024-67165-9

6. Liu K, Guo Q, Ding Y, Luo L, Huang J, Zhang Q. Alterations in nasal microbiota of patients with amyotrophic lateral sclerosis. *Chin Med J (Engl)*. Jan 20 2024;137(2):162-171. doi:10.1097/cm9.0000000000002701

7. De Jesus-Morales K, De Jesús-Rojas W, Ramos-Benitez MJ. Neutrophil-to-Lymphocyte Ratio Dynamics From Pre-diagnosis to End-Stage Amyotrophic Lateral Sclerosis (ALS): A Case Study on Association With Progression and Clinical Events. *Cureus*. 2024/4/12 2024;16(4):e58109. doi:10.7759/cureus.58109

8. Cao W, Cao Z, Tian Y*, et al*. Neutrophils Are Associated with Higher Risk of Incident Amyotrophic Lateral Sclerosis in a BMI- and Age-Dependent Manner. *Ann Neurol*. Nov 2023;94(5):942-954. doi:10.1002/ana.26760

9. Cotet C, Alarcan H, Hérault O*, et al*. Neutrophil to Lymphocyte Ratio as a Prognostic Marker in Amyotrophic Lateral Sclerosis. *Biomolecules*. Nov 23 2023;13(12)doi:10.3390/biom13121689

10. Grassano M, Manera U, De Marchi F*, et al*. The role of peripheral immunity in ALS: a population-based study. *Ann Clin Transl Neurol*. Sep 2023;10(9):1623-1632. doi:10.1002/acn3.51853

11. Leone MA, Mandrioli J, Russo S*, et al*. Neutrophils-to-Lymphocyte Ratio Is Associated with Progression and Overall Survival in Amyotrophic Lateral Sclerosis. *Biomedicines*. Feb 1 2022;10(2)doi:10.3390/biomedicines10020354

12. Wei QQ, Hou YB, Zhang LY*, et al*. Neutrophil-to-lymphocyte ratio in sporadic amyotrophic lateral sclerosis. *Neural Regen Res*. Apr 2022;17(4):875-880. doi:10.4103/1673-5374.322476

13. Cui C, Ingre C, Yin L*, et al*. Correlation between leukocyte phenotypes and prognosis of amyotrophic lateral sclerosis. *eLife*. 2022/03/15 2022;11:e74065. doi:10.7554/eLife.74065

14. Santos KdF. *Neutrophil-Lymphocyte Ratio (NLR) and Platelet-Lymphocyte Ratio (PLR) as neuroinflammatory biomarkers in Amyotrophic Lateral Sclerosis (ALS) progression*. 2022.

15. Li J-Y, Sun X-H, Shen D-c, Yang X-Z, Liu M-S, Cui L-Y. Clinical characteristics and prognosis of amyotrophic lateral sclerosis with autoimmune diseases. *PLOS ONE*. 2022;17(4):e0266529. doi:10.1371/journal.pone.0266529

16. Choi SJ, Hong YH, Kim SM, Shin JY, Suh YJ, Sung JJ. High neutrophil-to-lymphocyte ratio predicts short survival duration in amyotrophic lateral sclerosis. *Sci Rep*. Jan 16 2020;10(1):428. doi:10.1038/s41598-019-57366-y

17. Keizman D, Rogowski O, Berliner S*, et al*. Low-grade systemic inflammation in patients with amyotrophic lateral sclerosis. *Acta Neurol Scand*. Jun 2009;119(6):383-9. doi:10.1111/j.1600-0404.2008.01112.x
